# Real-world analysis of interstitial lung disease/pneumonitis in Japanese patients with breast cancer receiving trastuzumab deruxtecan

**DOI:** 10.1007/s12282-025-01814-3

**Published:** 2026-01-31

**Authors:** Junji Tsurutani, Kengo Noguchi, Ayumi Tanabe

**Affiliations:** 1 Advanced Cancer Translational Research Institute, Showa Medical University, 1‑5‑8, Hatanodai, Shinagawa‑ku, Tokyo, 142‑8666 Japan; 2 Pharmacoepidemiology & PMS Department, Daiichi Sankyo Co., Ltd., Tokyo, 103-8426 Japan; 3 Data Intelligence Department, Daiichi Sankyo Co., Ltd., Tokyo, 140-8710 Japan

**Keywords:** Breast cancer, Interstitial lung disease, Postmarketing surveillance, Real world, Trastuzumab deruxtecan

## Abstract

**Background:**

While trastuzumab deruxtecan (T-DXd) shows efficacy in various cancers, interstitial lung disease (ILD) and pneumonitis are significant risks associated with its use. Understanding these risks is crucial for effective management and safe administration of T-DXd. Therefore, we conducted a postmarketing surveillance study in Japan to investigate the incidence of ILD and related factors.

**Methods:**

All patients with human epidermal growth factor receptor 2-positive unresectable or recurrent breast cancer who initiated T-DXd in Japan between May 25, 2020, and November 30, 2021, were included. The primary outcome was the incidence of adjudicated drug-related ILD, and its associated factors were investigated using a Cox proportional hazards model.

**Results:**

The safety analysis set included 1,731 patients with a median (range) age of 60 (27–87) years. The median (range) duration of T-DXd treatment was 9.4 (0.7–17.9) months. A total of 24 (1.4%) and 11 (0.6%) patients had ILD as a medical history and comorbidity, respectively. The incidence of any Grade, Grade ≥ 3, and Grade 5 adjudicated drug-related ILD was 16.1%, 3.0%, and 1.0%, respectively. The median (range) time from the first T-DXd treatment to the first onset of ILD was 5.1 (0.5–17.3) months. Male sex, higher body mass index (≥ median [21.3 kg/m^2^]), history/comorbidity of ILD, and impaired renal function were identified as baseline factors potentially associated with an increased risk of drug-related ILD.

**Conclusions:**

The incidence of adjudicated drug-related ILD in the real-world setting was similar to that observed in clinical trials.

**Supplementary Information:**

The online version contains supplementary material available at 10.1007/s12282-025-01814-3.

## Introduction

In 2022, breast cancer was the most common cancer in Japanese women, constituting 21.6% of all cancers and ranking as the sixth leading cause of death overall (4.1% of all cancer-related deaths) [1]. Human epidermal growth factor receptor 2 (HER2) overexpression or amplification in patients with breast cancer is associated with poor prognosis and increased risk of disease recurrence and mortality compared with patients whose tumors do not overexpress HER2 [2, 3]. In the Japanese National Clinical Database (*N* = 95,620), 11.4% of patients had HER2-positive breast cancer in 2018 [4]. The introduction of the anti-HER2 monoclonal antibody trastuzumab revolutionized the management of HER2-positive breast cancer, improving the prognosis and survival of patients [5]. Subsequently, several drugs targeting the HER2 pathway have been approved for the treatment of HER2-positive breast cancer in Japan, including trastuzumab deruxtecan (T-DXd), which was approved in March 2020 to treat unresectable or recurrent HER2-positive breast cancer that has progressed after cancer chemotherapy [6]. The DESTINY-Breast01 trial showed an overall response rate (ORR) of approximately 61.0% in patients with metastatic breast cancer who had received a median of 6 prior treatments [7]. In subsequent clinical trials, T-DXd demonstrated a significantly longer progression-free survival (PFS) than physicians’ choice of chemotherapy (DESTINY-Breast02) [8] and trastuzumab emtansine (T-DM1) (DESTINY-Breast03) [9]. In the Phase 3, randomized, two-group, open-label, DESTINY-Breast04 trial involving patients with HER2-low metastatic breast cancer, T-DXd showed significantly longer PFS and overall survival than physicians’ choice of chemotherapy [10]. According to the 2022 Japanese Breast Cancer Society Clinical Practice Guidelines, T-DXd is a prioritized option recommended as a second-line treatment for patients with HER2-positive metastatic breast cancer that has progressed during or after combination therapy with trastuzumab, pertuzumab, and chemotherapy [6, 11].

Although T-DXd has greatly improved efficacy in HER2-positive breast cancer, interstitial lung disease (ILD)/pneumonitis has been identified as a potentially life-threatening adverse event (AE) of special interest [12]. ILD is a heterogeneous group of > 200 lung disorders, which manifest as inflammation and/or fibrosis of the lungs [13, 14]. Drug-related ILD has been observed in association with many anticancer agents, with different incidence and imaging patterns on computed tomography (CT) scans [15]. Notably, several reports have indicated that drug-related ILD has a higher incidence in Japan [14–16]. The incidence of adjudicated drug-related ILD by an ILD adjudication committee (ILD-AC) in the DESTINY-Breast01, -02, and -03 trials was 13.6%, 10%, and 15%, respectively, with most events being Grade 1–2; however, there were up to 2.2% of Grade 5 events [7–9]. The incidence of ILD (any Grade) in a pooled analysis of 9 T-DXd trials for different cancer types (*N* = 1,150; breast cancer: 44.3%) was 15.4% [17]. Similar to the findings of the DESTINY-Breast trials, > 75% of ILD events were low grade (Grade 1 or 2) [17]. In another systematic review and meta-analysis of 15 trials of T-DXd in patients with breast cancer (*N* = 1,970) and at a mean follow-up of 13.3 months, the incidence of ILD was 11.7% [18]. Thus, there is variability in the incidence of ILD across different reports. Given the above findings, ILD has been listed as an important identified risk in the Japanese risk-management plan for T-DXd, and the Japanese labeling for T-DXd states several risk-minimization measures, including tests for the regular monitoring of percutaneous oxygen saturation (SpO_2_), chest X-ray scans, and chest CT scans [19]. In addition, the 9-study pooled analysis reported the following potential clinical factors of interest for the development of T-DXd–related ILD: age < 65 years, enrollment in Japan, presence of lung comorbidities, moderate/severe renal impairment, time since initial diagnosis > 4 years, T-DXd dose > 6.4 mg/kg, and baseline SpO_2_ < 95% [17].

Diagnosing ILD requires excluding other respiratory diseases [13]. Its nonspecific symptoms often delay diagnosis, which can lead to severe ILD (Grade ≥ 3) [13]. It is crucial to investigate the risk of ILD to help ensure the appropriate use of T-DXd and ameliorate its risk for treatment-related side effects. The risk factors of T-DXd–related ILD have not been elucidated in a real-world setting. Given that the risk of ILD in the Japanese population is increased/higher compared with that in other parts of the world, further investigation of ILD in the real-world setting within a Japanese cohort is warranted. Thus, the objective of this postmarketing surveillance (PMS) study was to examine the real-world incidence of T-DXd–related ILD and identify the factors associated with its development in Japan.

## Patients and methods

### Study design and eligibility criteria

All patients with HER2-positive unresectable or recurrent breast cancer who initiated T-DXd in Japan between May 2020 and November 2021 were included in this study (jRCT1080225197). The observation period was 18 months based on the time to onset of ILD in previous clinical trials of T-DXd, including DESTINY-Breast01 [7], or until the end of the treatment for any reason, whichever came first. According to the ordinance of the Ministry of Health, Labour and Welfare No. 171, neither acquisition of informed consent from patients nor approval from the ethics committee at the participating sites was required for this PMS study. The study protocol was reviewed by the Pharmaceuticals and Medical Devices Agency in Japan.

### Outcomes and assessments

The safety analysis set included all patients whose case report forms (CRFs) had been collected, but excluded those who met the following criteria: did not receive T-DXd treatment; received the first dose of T-DXd after November 30, 2021; duplicate registration within the same site (if a patient was registered to > 1 site by site transfer, the patient was not excluded and counted as a different patient for each site); presence/absence of ILD could not be evaluated (no visit after the first visit); off-label use; prior treatment with T-DXd; and the study site did not agree to provide data for publication.

The effectiveness analysis set included all patients in the safety analysis set except those in whom the T-DXd dose deviated from the approved dosage and administration and who did not have effectiveness assessment data.

The primary outcome of interest was the incidence of adjudicated drug-related ILD. All potential ILD events that physicians reported in the CRFs, where the possibility of ILD could not be ruled out, were adjudicated by an independent ILD-AC. ILD adjudication was performed with access to relevant clinical information in an unblinded manner. The ILD-AC comprised radiologists, pulmonologists, breast cancer experts, and gastric cancer experts [20]. Treatment-emergent ILD was defined as ILD that occurred, having been absent before the first dose of T-DXd, or worsened in severity after the first dose of T-DXd. If an ILD occurred, information about the ILD, including the outcome, was assessed for up to a maximum of 6 months (26 weeks) after ILD onset.

ILD that recurred after rechallenge with T-DXd (T-DXd should be discontinued if ILD occurs; however, it could have been administered again based on the investigator’s decision) was not included in the analysis and was listed separately. If there was > 1 outcome on the latest outcome date, 1 outcome was selected in the following order: fatal, unknown, not recovered, recovered with sequelae, recovering, and recovered. Severity assessment of ILD grades was based on the Japanese translation of the National Cancer Institute Common Terminology Criteria for Adverse Events version 5.0 [21]. Assessment of ILD imaging patterns was conducted by multiple radiologists who retrospectively reviewed chest CT images from adjudicated drug-related ILD using a process similar to that in the T-DXd clinical trials. The images were classified into the following categories: diffuse alveolar damage (DAD) pattern, organizing pneumonia (OP) pattern, hypersensitivity pneumonitis (HP) pattern, nonspecific interstitial pneumonia (NSIP) pattern, and others.

The effectiveness outcomes included the ORR and best ORR according to the Response Evaluation Criteria in Solid Tumors version 1.1 for both systemic and intracranial tumors [22].

### Statistical analyses

The incidence of ILD in the HER2-positive breast cancer 5.4 mg/kg pooled population in the clinical trials of T-DXd was 22/234 (9.4%) in the overall population, which included participants from various countries and regions (unpublished information), and 10/51 (19.6%) in the Japanese subgroup [19]. It was assumed that the incidence of ILD in routine clinical practice would be ≥ 10%, and accordingly, 1,500 patients were to be enrolled in the PMS to conduct an analysis of risk factors in patients with ILD with appropriate statistical power.

The confidence coefficient of the confidence interval (CI) was 95%. The factors associated with the development of adjudicated drug-related ILD were investigated using a Cox proportional hazards model. The factors considered in the multivariable analysis included exploratory factors that demonstrated significant differences in the univariable analyses, as well as potential risk factors for ILD determined a priori based on medical rationale: the Eastern Cooperative Oncology Group performance status (ECOG PS), lung/pleural metastasis or recurrence, serum albumin, SpO_2_, and renal function. The variables that showed significance in the multivariable analysis were subjected to medical assessment to identify factors of interest associated with ILD development. Results of the univariable and multivariable analyses are presented as hazard ratios (HRs) and 95% CIs. All statistical analyses were performed using SAS^®^ System Release 9.4 (SAS Institute, Cary, NC, USA).

## Results

### Patient disposition and treatment status

**Fig. 1:**
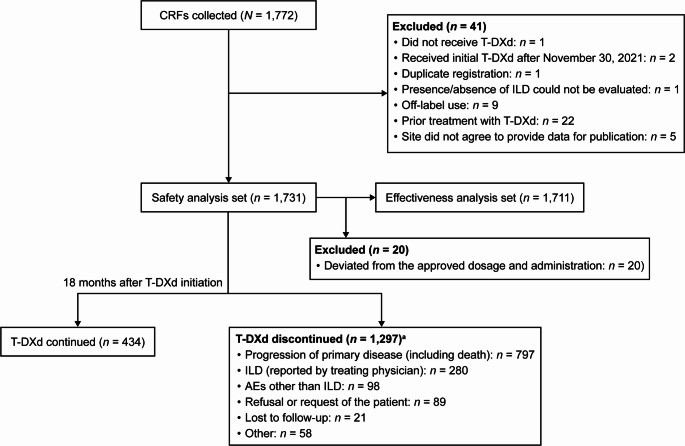
Patient disposition and treatment status. ^a^Categories were not mutually exclusive.* AE* adverse event;* CRF* case report form;* ILD* interstitial lung disease;* T-DXd* trastuzumab deruxtecan

CRFs from 1,772 patients were collected; after exclusions, 1,731 and 1,711 patients were included in the safety and effectiveness analysis set, respectively (Fig. 1). Eighteen months after the initiation of T-DXd, 434 patients continued T-DXd treatment, and 1,297 patients had discontinued treatment. Reasons for treatment discontinuation included disease progression (46.0%), AEs other than ILD (5.7%), ILD reported by the investigator (16.2%), lost to follow-up (1.2%), patient refusal (5.1%), and other causes (3.4%).

### Demographics and baseline clinical characteristics

In the safety analysis set (*n* = 1,731), 1,723 (99.5%) patients were female, and the median (range) age was 60 (27–87) years (Table 1). The ECOG PS was 0–1 in 1,594 (92.1%) patients, the median body mass index (BMI) was 21.3 kg/m^2^, and 1,467 (84.7%) patients had stage IV unresectable or recurrent breast cancer. The time from recurrent or unresectable breast cancer diagnosis was ≥ 48 months in 709 (41.0%) patients and ≥ 24 to < 48 months in 515 (29.8%) patients. The most common sites of metastasis or recurrence (including multiple locations) were the local lymph nodes in 1,055 (60.9%) patients; 894 (51.6%) patients had lung/pleural metastasis, and 367 (21.2%) patients had brain metastasis. Most patients had normal or mild impairment in renal (79.3%) and hepatic (96.9%) functions. A total of 24 (1.4%) patients had a history of ILD, and 11 (0.6%) patients had ILD at baseline as a comorbidity. The median (range) of prior treatment regimens for unresectable or recurrent breast cancer was 4 (0–39), and > 90% of patients had received ≥ 2 prior treatment regimens, whereas 99.1% had received prior anti-HER2 therapies.

**Table 1:** Demographics and baseline clinical characteristics (safety analysis set)

	Patients (*N* = 1,731)
Sex, *n* (%)	
Male	8 (0.5)
Female	1,723 (99.5)
Age, median (range), years	60 (27–87)
< 65, *n* (%)	1,094 (63.2)
≥ 65, *n* (%)	637 (36.8)
BMI (kg/m^2^), median (range)	21.3 (12.9–39.8)
History of smoking, *n* (%)	
Never smoked	1,257 (72.6)
Past smoker	195 (11.3)
Current smoker	34 (2.0)
Unknown/missing	245 (14.2)
ECOG PS, *n* (%)	
0–1	1,594 (92.1)
2–4	137 (7.9)
Time from recurrent or unresectable breast cancer diagnosis (months), *n* (%)	
< 6	59 (3.4)
≥ 6 to < 12	111 (6.4)
≥ 12 to < 24	317 (18.3)
≥ 24 to < 48	515 (29.8)
≥ 48	709 (41.0)
Unknown/missing	20 (1.2)
Stage of unresectable or recurrent breast cancer, *n* (%)	
IIIB	38 (2.2)
IIIC	64 (3.7)
IV	1,467 (84.7)
Other	161 (9.3)
Unknown/missing	1 (0.1)
Site(s) of metastasis or recurrence^a^, *n* (%)	
None	3 (0.2)
Local/lymph node	1,055 (60.9)
Lung/pleural	894 (51.6)
Liver	609 (35.2)
Brain	367 (21.2)
Meninges/spinal fluid	14 (0.8)
Bone	744 (43.0)
Other	212 (12.2)
Renal function (CrCl^b^ [mL/min]),*n* (%)	
Normal: ≥ 90	605 (35.0)
Mild impairment: ≥ 60 to < 90	767 (44.3)
Moderate impairment: ≥ 30 to < 60	313 (18.1)
Severe impairment: ≥ 15 to < 30	12 (0.7)
End-stage renal disease: < 15	7 (0.4)
Unknown/missing	27 (1.6)
Hepatic function (TB [mg/dL] and AST [IU/L]), *n* (%)	
Normal: TB ≤ 1.5 and AST ≤ 30	673 (38.9)
Mild impairment: TB > 1.5 to ≤ 2.25 or AST > 30	1,005 (58.1)
Moderate impairment: TB > 2.25 to ≤ 4.5	15 (0.9)
Severe impairment: TB > 4.5	6 (0.3)
Unknown/missing	32 (1.8)
SpO_2_ (%),*n* (%)	
< 95	51 (2.9)
≥ 95	1,297 (74.9)
Not implemented	369 (21.3)
Unknown/missing	14 (0.8)
Medical history, *n* (%)	
Respiratory disease	104 (6.0)
ILD	24 (1.4)
Radiation pneumonitis	40 (2.3)
COPD or emphysema	1 (0.1)
Asthma	29 (1.7)
Other respiratory disease	15 (0.9)
History of lung surgery	76 (4.4)
Malignant tumors other than breast cancer	58 (3.4)
Comorbidities at baseline, *n* (%)	
Respiratory disease	87 (5.0)
ILD	11 (0.6)
Radiation pneumonitis	31 (1.8)
COPD or emphysema	4 (0.2)
Asthma	23 (1.3)
Other respiratory disease	22 (1.3)
Pleural effusion	230 (13.3)
Malignant tumors other than breast cancer	26 (1.5)
Prior cancer therapy for unresectable or recurrent breast cancer, *n* (%)	
No	8 (0.5)
Yes	1,720 (99.4)
Median (range)	4 (0–39)
Unknown/missing	3 (0.2)
Anti-HER2 therapies, *n* (%)	
No	13 (0.8)
Yes	1,715 (99.1)
Trastuzumab	1,615 (93.3)
Pertuzumab	1,537 (88.8)
Trastuzumab emtansine	1,654 (95.6)
Lapatinib	554 (32.0)
Other	0 (0.0)
Unknown/missing	3 (0.2)
Molecular targeted therapies, *n* (%)	
No	1,496 (86.4)
Yes	232 (13.4)
Bevacizumab	168 (9.7)
Everolimus	26 (1.5)
Palbociclib	53 (3.1)
Abemaciclib	31 (1.8)
Palbociclib or abemaciclib	74 (4.3)
Other	6 (0.3)
Unknown/missing	3 (0.2)
Immune checkpoint inhibitors, *n* (%)	
None	1,726 (99.7)
Any	2 (0.1)
Unknown/missing	3 (0.2)
Chemotherapy, *n* (%)	
No	171 (9.9)
Yes	1,557 (89.9)
Taxane (docetaxel, paclitaxel, nab-paclitaxel)	1,304 (75.3)
Anthracycline (epirubicin, doxorubicin)	305 (17.6)
Eribulin	641 (37.0)
Gemcitabine	190 (11.0)
Vinorelbine	374 (21.6)
Capecitabine	616 (35.6)
Other	237 (13.7)
Unknown/missing	3 (0.2)
Other, *n* (%)	
No	1,714 (99.0)
Yes	14 (0.8)
Unknown/missing	3 (0.2)

*AST* aspartate aminotransferase;* BMI* body mass index;* COPD* chronic obstructive pulmonary disease;* CrCl* creatinine clearance;* ECOG PS* Eastern Cooperative Oncology Group performance status;* HER2* human epidermal growth factor receptor 2;* ILD* interstitial lung disease;* SpO*_2_ oxygen saturation;* TB* total bilirubin

^a^Including metastases or recurrence in multiple locations

^b^Calculated using the Cockcroft-Gault equation

### T-DXd dosage by treatment cycle

The median (range) duration of T-DXd treatment was 9.4 (0.7–17.9) months. Throughout the treatment period, most patients received > 4.4 to ≤ 5.4 mg/kg of T-DXd (Online Resource Supplementary Fig. 1). No patient received T-DXd beyond the approved dosage (5.4 mg/kg).

### Incidence of adjudicated drug-related ILD

**Fig. 2:**
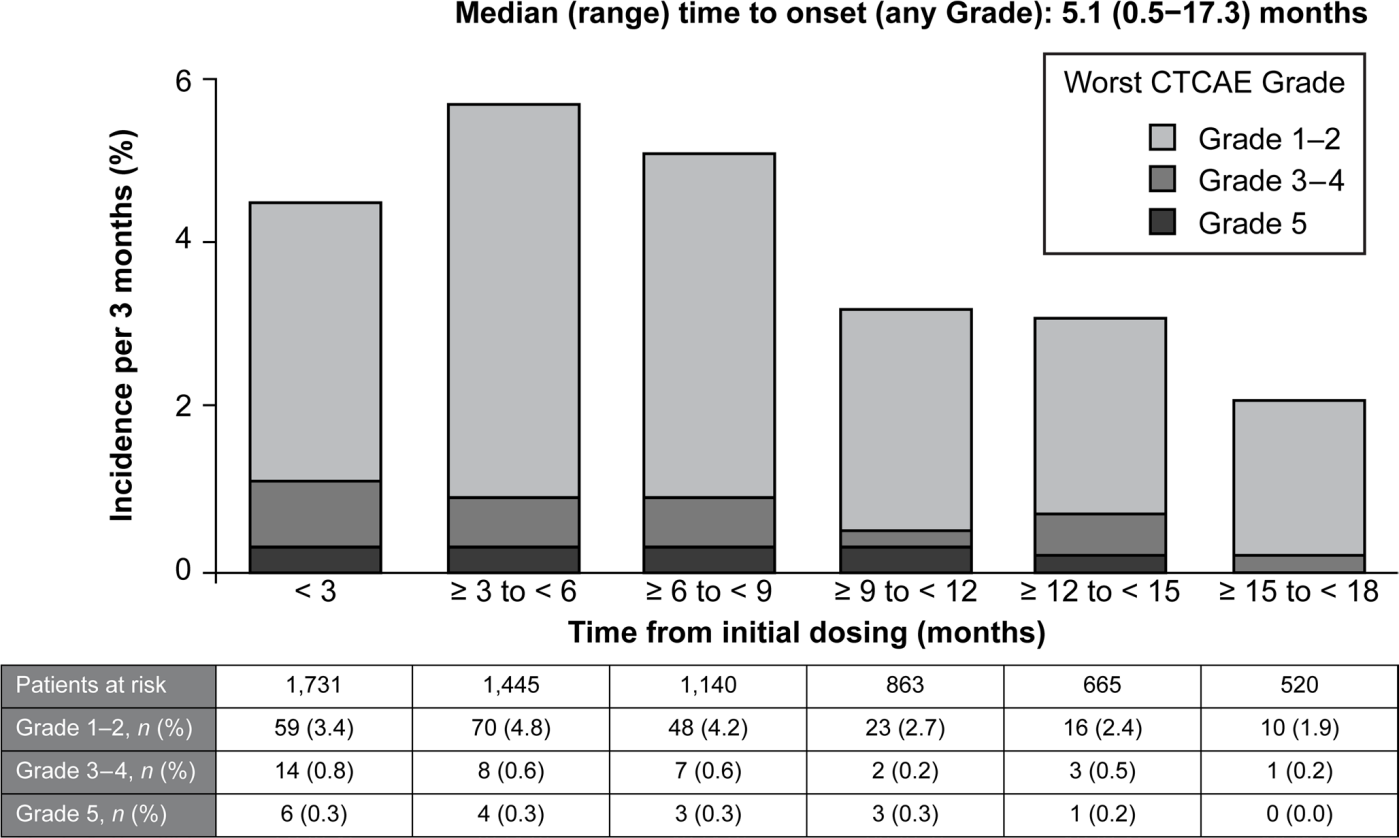
Time to onset of adjudicated drug-related ILD. All potential ILD events reported by physicians, for which ILD could not be excluded, were reviewed by an independent ILD adjudication committee. *CTCAE* Common Terminology Criteria for Adverse Events; *ILD* interstitial lung disease

The incidence of any Grade, Grade ≥ 3, and Grade 5 adjudicated drug-related ILD was 16.1% (278/1,731), 3.0% (52/1,731), and 1.0% (17/1,731), respectively (Table 2). The median (range) time from the first T-DXd treatment to the first onset of ILD episode was 5.1 (0.5–17.3) months. The median (range) time to ILD Grade ≥ 3 and Grade 5 onset was 4.6 (0.5–15.9) months and 5.6 (0.5–13.2) months, respectively. Adjudicated drug-related ILD was reported throughout the 18-month observation period (Fig. 2). The incidence of any Grade adjudicated drug-related ILD was the highest during ≥ 3 to < 6 months (5.7%) and that of Grade ≥ 3 adjudicated drug-related ILD was the highest during < 3 months (1.2%) of T-DXd treatment, whereas the development of adjudicated drug-related ILD was observed throughout the observation period.

**Table 2:** Incidence of adjudicated drug-related ILD

Worst CTCAE grade	*n* (%)
Total	1,731
Any Grade	278 (16.1)
Grade 1	125 (7.2)
Grade 2	101 (5.8)
Grade 3	34 (2.0)
Grade 4	1 (0.1)
Grade 5	17 (1.0)
Grade ≥ 3	52 (3.0)

*CTCAE* Common Terminology Criteria for Adverse Events;* ILD* interstitial lung disease

### Imaging pattern at onset, and treatment and outcome of adjudicated drug-related ILD

The most common imaging pattern at onset of any Grade adjudicated drug-related ILD (*N* = 278) was OP (*n* = 195, 70.1%), followed by HP (*n* = 44, 15.8%), DAD (*n* = 26, 9.4%), and NSIP (*n* = 5, 1.8%) (Online Resource Supplementary Table 1). Among patients with Grade 1–2, Grade 3–4, and Grade 5 adjudicated drug-related ILD, the most common imaging pattern was OP (174/226, 77.0%), OP (18/35, 51.4%), and DAD (12/17, 70.6%), respectively. A total of 45.7% of patients with any Grade ILD did not receive any treatment for ILD (Table 3). All patients with Grade ≥ 3 ILD received steroids, with 71.2% receiving steroid pulse therapy (defined as methylprednisolone ≥ 500 mg/day). The combined outcome of any Grade ILD was recovered, recovering, or recovered with sequelae in > 80% of patients up to 6 months of follow-up after the onset of adjudicated drug-related ILD (Table 4). For Grade ≥ 3 ILD (*n* = 52), 5 (9.6%) patients did not recover within the observation period and 17 (32.7%) were fatal. Among patients with Grade 5 adjudicated drug-related ILD (*n* = 17), 2 had a history of ILD, 1 had a history of both ILD and lung surgery, and 1 had radiation pneumonitis (Online Resource Supplementary Table 2). The time from ILD onset to death was 3–370 days. Except for 1 patient with Grade 5 ILD, all others received steroid pulse.

**Table 3:** Treatment for adjudicated drug-related ILD

Worst CTCAE grade	Adjudicated drug-related ILD, *N*	Treatment, *n* (%)	Details of action taken^a^, *n* (%)				
		None	Any	Steroid (including steroid pulse)^b^	Steroid pulse^b^	Immunosuppressants	Others
Any Grade	278	127 (45.7)	151 (54.3)	147 (52.9)	45 (16.2)	1 (0.4)	16 (5.8)
Grade 1	125	104 (83.2)	21 (16.8)	18 (14.4)	0 (0.0)	0 (0.0)	3 (2.4)
Grade 2	101	23 (22.8)	78 (77.2)	77 (76.2)	8 (7.9)	0 (0.0)	1 (1.0)
Grade 3	34	0 (0.0)	34 (100.0)	34 (100.0)	20 (58.8)	0 (0.0)	6 (17.6)
Grade 4	1	0 (0.0)	1 (100.0)	1 (100.0)	1 (100.0)	0 (0.0)	0 (0.0)
Grade 5	17	0 (0.0)	17 (100.0)	17 (100.0)	16 (94.1)	1 (5.9)	6 (35.3)
Grade ≥ 3	52	0 (0.0)	52 (100.0)	52 (100.0)	37 (71.2)	1 (1.9)	12 (23.1)

*CTCAE* Common Terminology Criteria for Adverse Events;* ILD* interstitial lung disease

^a^Categories are not mutually exclusive

^b^Defined as methylprednisolone ≥ 500 mg/day

**Table 4:** Outcome of adjudicated drug-related ILD

Worst CTCAE grade	Adjudicated drug-related ILD, *N*	Outcome (up to 6 months of follow-up after onset of ILD), *n* (%)					
		Recovered	Recovering	Recovered with sequelae	Not recovered	Fatal	Unknown/missing
Any Grade	278	146 (52.5)	69 (24.8)	15 (5.4)	26 (9.4)	17 (6.1)	5 (1.8)
Grade 1	125	78 (62.4)	28 (22.4)	0 (0.0)	15 (12.0)	0 (0.0)	4 (3.2)
Grade 2	101	60 (59.4)	29 (28.7)	5 (5.0)	6 (5.9)	0 (0.0)	1 (1.0)
Grade 3	34	8 (23.5)	12 (35.3)	9 (26.5)	5 (14.7)	0 (0.0)	0 (0.0)
Grade 4	1	0 (0.0)	0 (0.0)	1 (100.0)	0 (0.0)	0 (0.0)	0 (0.0)
Grade 5	17	NA	NA	NA	NA	17 (100.0)	NA
Grade ≥ 3	52	8 (15.4)	12 (23.1)	10 (19.2)	5 (9.6)	17 (32.7)	0 (0.0)

*CTCAE* Common Terminology Criteria for Adverse Events;* ILD* interstitial lung disease;* NA* not applicable

### Factors of interest for the development of ILD

**Fig. 3:**
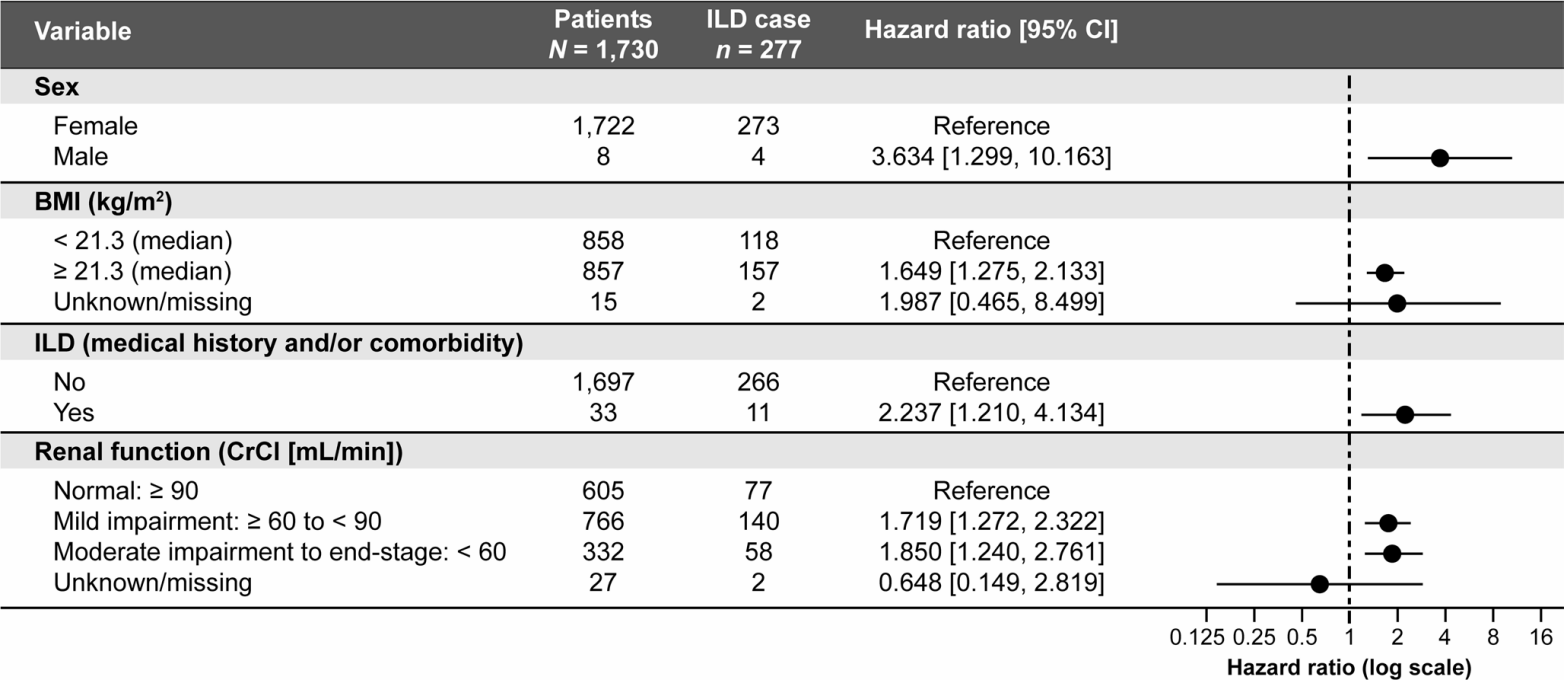
Forest plot of factors of interest from multivariable Cox regression analysis^a^ for the development of adjudicated drug-related ILD. ^a^See Online Resource Supplementary Table 4.* BMI* body mass index;* CI* confidence interval;* CrCl* creatinine clearance;* ILD* interstitial lung disease

In the univariable analyses, male sex, age ≥ 65 years, BMI ≥ 21.3 kg/m^2^ (the median value of the population), presence of lung/pleural metastasis or recurrence, presence of liver metastasis or recurrence, medical history and/or comorbidity of ILD or pleural effusion, history of lung surgery, and presence of renal impairment were associated with a significantly increased risk of development of ILD (Online Resource Supplementary Table 3). In the multivariable analysis, male patients had a significantly increased risk for developing ILD compared with female patients (HR [95% CI]: 3.634 [1.299–10.163]) (Fig. 3 and Online Resource Supplementary Table 4). Patients with BMI ≥ 21.3 kg/m^2^ (HR [95% CI]: 1.649 [1.275–2.133]), medical history and/or comorbidity of ILD at baseline (HR [95% CI]: 2.237 [1.210–4.134]), and renal impairment (mild impairment vs. no impairment, HR [95% CI]: 1.719 [1.272–2.322] and moderate impairment to end-stage impairment, HR [95% CI]: 1.850 [1.240–2.761]) had a significantly increased risk of the development of ILD.

### Effectiveness

The ORR was 60.5% (complete response [CR]: 5.7% and partial response [PR]: 54.8%) in the overall effectiveness analysis set. The intracranial ORR in the subgroup of patients with brain metastasis was 36.4% (CR: 8.5% and PR: 27.9%) (Online Resource Supplementary Table 5).

## Discussion

PMS studies are important to evaluate the performance of a drug in routine clinical practice settings, where strict exclusion criteria, such as those used in clinical trials, are not applied. The present PMS study included > 1,700 patients with HER2-positive unresectable or recurrent breast cancer who received T-DXd in Japan since its launch in May 2020, thereby accurately reflecting the real-world treatment patterns and safety of T-DXd in Japan. The incidence of adjudicated drug-related ILD was 16.1%, with > 80% of events being Grade 1–2 and > 80% with an outcome of recovered, recovering, or recovered with sequelae. The incidence of fatal (Grade 5) adjudicated drug-related ILD (1.0%) in the PMS study did not exceed that described in the pooled analysis of ILD from the clinical trials for HER2-positive breast cancer treated with T-DXd 5.4 mg/kg every 3 weeks (2.9%) [17]. The findings of this study suggest that the risk of ILD in the real-world setting was similar to that reported in clinical trials. This could be attributed to the efforts that adhere to ILD management guidelines, including risk-minimization measures [23].

This PMS study included patients who were ineligible for participation in DESTINY-Breast trials, such as 7.9% with an ECOG PS ≥ 2, 1.1% with pre-existing severe renal impairment (creatinine clearance < 30 mL/min), and 8.7% with a history or presence of pulmonary conditions, ILD, radiation pneumonitis, chronic obstructive pulmonary disease (COPD), emphysema, or asthma [7–9]. The recommended dose of T-DXd for breast cancer is 5.4 mg/kg every 3 weeks [19]. In the present study, most patients received > 4.4 to ≤ 5.4 mg/kg T-DXd across treatment cycles. While a substantial proportion of patients received T-DXd at the approved dosage (5.4 mg/kg), the dosage was reduced by some physicians based on the patient’s condition including the initial dosing. Thus, this study revealed that T-DXd is also used in patients ineligible for clinical trials and that the initial dosage was reduced in some patients in the real-world setting.

ILD is a well-described adverse drug reaction associated with several anti-HER2 drugs, with an incidence of up to 1.8% (lapatinib-based therapy) to 13.6%–17.4% for T-DXd [24]. In line with several reports indicating that drug-related ILD has a higher incidence in Japan [14–16], a subgroup analysis of the Asian population in the DESTINY-Breast03 trial revealed that the incidence of adjudicated T-DXd–related ILD was higher in the Japanese population than in the non-Japanese Asian and global populations [25]. Furthermore, a large retrospective real-world study in French patients with HER2-driven breast cancer and other malignancies reported a T-DXd–related ILD incidence of 11.2% [26]. Based on the above findings, the incidence of adjudicated drug-related ILD in the present study (16.1%) was comparable to that demonstrated in the DESTINY-Breast trials (10.0%–15.0%) [7–9], highlighting that the difference in the incidence of adjudicated T-DXd–related ILD between the Japanese and global populations is observed not only in clinical trials but also in real-world settings. The quarterly incidence of adjudicated drug-related ILD was higher during the first 9 months than during the last 9 months after the initial T-DXd dosing; in fact, in this PMS study, most ILD cases, regardless of their worst grade, were reported within the first 9 months of treatment. However, adjudicated drug-related ILD can occur throughout the treatment period, and continuous risk management for ILD is necessary throughout the entire treatment duration. ILD can present with different radiographic disease patterns, including OP, HP, and DAD, with the DAD pattern being associated with a higher risk of mortality [13, 20, 27]. In the present study, assessment of the imaging pattern at onset revealed OP, HP, and NSIP in > 80% (244/278) of patients with ILD. In contrast, DAD was seen in approximately 50% of patients each with Grade 3–4 and Grade 5 ILD. The outcome for > 80% of any Grade ILD in the present study was recovered, recovering, or recovered with sequelae, while the proportion decreased to < 60% in the case of Grade ≥ 3. It should be noted that the presenting outcomes are based on observations up to 6 months after the onset of adjudicated drug-related ILD.

In the multivariable analysis, the following variables were identified as factors of interest for the development of ILD: male sex, BMI ≥ median (≥ 21.3 kg/m^2^), history of ILD, and renal impairment.

Although breast cancer mostly occurs in women, 0.6% of patients were diagnosed with male breast cancer between January 2012 and December 2018 in the Japanese National Clinical Database (*N* = 594,316) [28]. Some studies have identified male sex as a risk factor for drug-induced ILD following treatment with panitumumab and pemetrexed in patients with colorectal cancer and malignant pleural mesothelioma/non-small cell lung cancer, respectively [29, 30]. The small number of male patients (*n* = 8) in this study limits interpretation. Further investigations are warranted to validate this finding.

In the present study, BMI ≥ median was associated with a significantly increased risk of ILD compared with BMI < median, suggesting that a higher BMI could predispose patients to the development of ILD. However, it should be noted that the cutoff used for BMI in the present study was not based on the conventional normal (≥ 18.5 to ≤ 24.9 kg/m^2^), overweight (≥ 25 to ≤ 29.9 kg/m^2^), and obese (≥ 30 kg/m^2^) categories [31], but on the median BMI value (21.3 kg/m^2^). In the univariable analyses, both conventional categories and categories determined by the median BMI were examined, and significant differences were observed only in the median-based categories. Based on this result, the multivariable analysis included only the categories determined by the median value. With BMI calculated as weight divided by height squared [31], the working hypothesis was that the weight and dosage of T-DXd were positively correlated, but the lung volume, which is correlated with height, was negatively correlated with the incidence of ILD. There are a few reports suggesting an association between obesity and ILD [32, 33], whereas a recent report indicates that patients with a higher BMI (> 25 kg/m²) experienced increased rates of T-DXd dose reduction and T-DXd–related toxicity compared with those with a lower BMI (< 25 kg/m²) [34]. This can be interpreted as indicating that patients with a higher BMI have a lower threshold for T-DXd–related toxicity, including ILD, which is in line with the findings of this study. The mean BMI of female patients aged ≥ 20 years in the general population was 29.8 kg/m^*2*^ in the United States [35] and 22.5 kg/m^*2*^ in Japan [36]. The lower BMI among the Japanese population compared with the US population may explain the impact of BMI observed in this study. As BMI and obesity are modifiable characteristics, once an association with the development of ILD is confirmed, appropriate management could serve as a preventive measure against the development of drug-related ILD.

Medical history and/or comorbidity of ILD at baseline was associated with a significantly increased risk for ILD compared with no medical history and/or comorbidity of ILD in the present study. In the pooled analysis of ILD in 9 T-DXd monotherapy clinical trials, pre-existing lung comorbidities (prior ILD, asthma, COPD, pulmonary fibrosis, pulmonary emphysema, and radiation pneumonitis) were identified as factors potentially associated with the development of ILD [17]. Medical history and/or comorbidity of ILD has also been recognized as an independent risk factor for drug-induced ILD across several anticancer medications [16, 37].

In the pooled analysis of T-DXd, moderate/severe renal impairment was identified as one of the factors of interest associated with the development of ILD [17]. In line with the previous report regarding clinical trials, the present study revealed that pre-existing mild renal impairment and moderate renal impairment to end-stage renal disease were associated with a significantly increased risk for the development of ILD compared with normal renal function. The possible risk of ILD in patients with moderate renal impairment is described in the United States package insert and European summary of product characteristics [38, 39]. There is a close physiological relationship between the kidneys and the lungs, and kidney diseases can cause lung damage [40]. One possible hypothesis is that baseline renal impairment may be linked to quiescent damage to the lungs before treatment with T-DXd, which could lower the threshold for the development of ILD [40]. Another possibility is that renal impairment may serve as a surrogate for patient status, which impacts the risk of ILD. Further investigation, including an analysis of pathophysiological mechanisms, is therefore necessary to understand and manage the risk of ILD in patients with renal impairment.

This study benefits from several key strengths. First, the inclusion of all patients with HER2-positive unresectable or recurrent breast cancer treated with T-DXd for a certain period after the launch resulted in a large-scale study; therefore, the results likely reflect the true clinical landscape in Japan. Second, the prospective cohort study design allowed for the collection of high-quality, longitudinal data. Third, the presence of ILD was determined by an independent ILD-AC, thereby minimizing variability in the assessment criteria for the event. Lastly, the study reflects the real-world situation in Japan, where the incidence of ILD has been higher than that in other regions. However, this could also be considered a limitation, as caution is warranted when generalizing these findings to populations outside of Japan. Another limitation is that the observation period of this study was only up to 18 months, which lacks long-term observation. Furthermore, the number of male patients was only 8, which may potentially lead to reduced confidence in the validity of this finding as an identified factor of interest. Finally, we acknowledge that potential reporting bias is inherent in PMS data.

## Conclusion

This is the first study to provide real-world patient experience with T-DXd in > 1,700 patients with HER2-positive breast cancer in Japan. The findings indicate that the risk of ILD in the real-world setting was similar to those observed in clinical trials.

## Supplementary Information

Below is the link to the electronic supplementary material.

Supplementary Material 1

## Data Availability

Data included in this manuscript are used under a contract with the participating site, but the provision of data is not included in the contract, and it cannot be freely distributed by the authors.
