# Supplementary material for: Real-world analysis of interstitial lung disease/pneumonitis in Japanese patients with breast cancer receiving trastuzumab deruxtecan
Source: Breast Cancer. 2026 Jan 31;33(2):346–58. doi: 10.1007/s12282-025-01814-3 (PMC12960362; doi:10.1007/s12282-025-01814-3)
Supplement: Supplementary file 1 — Supplementary Material 1 [file 12282_2025_1814_MOESM1_ESM.docx]

**SUPPLEMENTARY MATERIAL**

**Real-world analysis of interstitial lung disease/pneumonitis in Japanese patients with breast cancer receiving trastuzumab deruxtecan**

Junji Tsurutani,^1^ Kengo Noguchi,^2^ Ayumi Tanabe^3^

^1^Advanced Cancer Translational Research Institute, Showa Medical University, Tokyo 142‑8666, Japan

^2^Pharmacoepidemiology & PMS Department, Daiichi Sankyo Co., Ltd., Tokyo 103-8426, Japan

^3^Data Intelligence Department, Daiichi Sankyo Co., Ltd., Tokyo 140-8710, Japan

**Corresponding author**

Junji Tsurutani

Advanced Cancer Translational Research Institute, Showa Medical University

1‑5‑8, Hatanodai, Shinagawa‑ku, Tokyo 142‑8666, Japan

Telephone number: +81-3-3784-8145

E-mail address: [tsurutaj@med.showa-u.ac.jp](mailto:tsurutaj@med.showa-u.ac.jp)

ORCiD: 0000-0002-6260-6943

**Online Resource Supplementary Fig. 1** T-DXd dose by treatment cycle


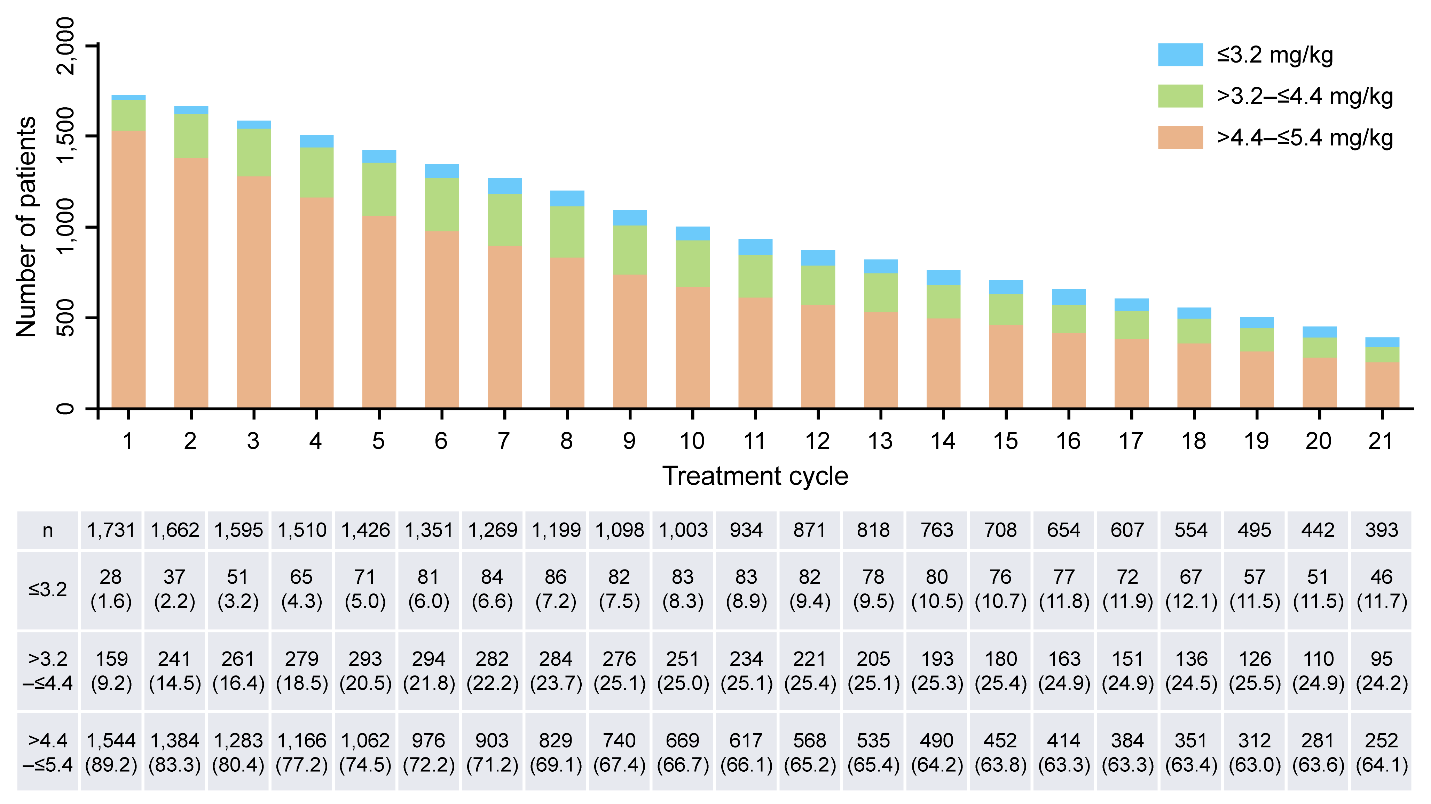


Data are presented as n (%)

T-DXd, trastuzumab deruxtecan

**Online Resource Supplementary Table 1** Imaging patterns at onset by worst CTCAE grade of adjudicated drug-related ILD (safety analysis set)

|  | **N** | **n (%)** | **n (%)** | **n (%)** |
| --- | --- | --- | --- | --- |
| **Imaging pattern** |  | **Grade 1–2** | **Grade 3–4** | **Grade 5** |
| All | 278 | 226 (81.3) | 35 (12.6) | 17 (6.1) |
| DAD | 26 | 2 (7.7) | 12 (46.2) | 12 (46.2) |
| HP | 44 | 39 (88.6) | 4 (9.1) | 1 (2.3) |
| OP | 195 | 174 (89.2) | 18 (9.2) | 3 (1.5) |
| NSIP | 5 | 4 (80.0) | 0 (0.0) | 1 (20.0) |
| Other/indeterminate | 4^a^ | 4 (100.0) | 0 (0.0) | 0 (0.0) |
| Not determined | 4^b^ | 3 (75.0) | 1 (25.0) | 0 (0.0) |

^a^Two cases with radiation recall, noncardiogenic pulmonary edema pattern, and faint ground‑glass opacity in the lower lobe of the left lung

^b^Two cases that could not be diagnosed due to very faint shadows, and 2 cases in which images were unavailable

CTCAE, Common Terminology Criteria for Adverse Events; DAD, diffuse alveolar damage; HP, hypersensitivity pneumonitis; ILD, interstitial lung disease; NSIP, nonspecific interstitial pneumonia; OP, organizing pneumonia

**Online Resource Supplementary Table 2** Details of patients with Grade 5 adjudicated drug-related ILD^a,b,c,d^ (safety analysis set)

| **Subject No. of ILD** | **Age (years)** | **Body weight (kg)** | **BMI (kg/m^2^)** | **ECOG PS** | **Recurrent or metastasis** | **Medical history** | **Comorbidity** | **Imaging pattern  (at onset)** | **CTCAE  grade  at onset** | **Time from initial dosing to ILD onset (days)** | **Time from ILD onset to death (days)** | **Other  treatment for ILD** | **Steroid pulse** |
| --- | --- | --- | --- | --- | --- | --- | --- | --- | --- | --- | --- | --- | --- |
| 1 | 65 | 35.0 | 15.6 | 1 | Recurrent | None | Pleural effusion | DAD | Grade 3 | 84 | 5 | Steroid Other | Yes |
| 2 | 68 | 52.2 | 22.0 | 0 | Recurrent | ILD | None | OP | Grade 3 | 252 | 69 | Steroid | Yes |
| 3 | 43 | 73.8 | 29.9 | 0 | Recurrent | None | None | DAD | Grade 3 | 121 | 51 | Steroid | Yes |
| 4 | 82 | 49.0 | 21.8 | 0 | Recurrent | None | None | DAD | Grade 2 | 205 | 45 | Steroid | Yes |
| 5 | 74 | 45.0 | 21.4 | 1 | Metastasis | None | None | DAD | Grade 2 | 175 | 33 | Steroid | Yes |
| 6 | 40 | 61.2 | 23.6 | 1 | Metastasis | None | None | OP | Grade 3 | 41 | 35 | Steroid Other | No |
| 7 | 68 | 40.0 | 20.4 | 2 | Metastasis | None | None | NSIP | Grade 1 | 154 | 52 | Steroid IS | Yes |
| 8 | 45 | 55.0 | 23.5 | 0 | Recurrent | ILD | Autoimmune thyroiditis | DAD | Grade 3 | 290 | 14 | Steroid Other | Yes |
| 9 | 76 | 39.0 | 16.4 | 1 | Recurrent | None | None | DAD | Grade 3 | 170 | 3 | Steroid | Yes |
| 10 | 71 | 37.5 | 17.1 | 1 | Recurrent | ILD History of lung surgery | Pleural effusion | DAD | Grade 3 | 35 | 11 | Steroid | Yes |
| 11 | 66 | 44.9 | 21.1 | 0 | Recurrent | Radiation Pneumonitis | Uterine cancer | DAD | Grade 3 | 209 | 13 | Steroid | Yes |
| 12 | 31 | 43.7 | 18.3 | 3 | Metastasis | None | Pleural effusion | OP | Grade 3 | 19 | 370 | Steroid | Yes |
| 13 | 70 | 64.0 | 27.1 | 1 | Recurrent | None | Pleural effusion | DAD | Grade 1 | 14 | 26 | Steroid | Yes |
| 14 | 60 | 59.0 | 25.2 | 1 | Recurrent | None | None | DAD | Grade 3 | 401 | 12 | Steroid Other | Yes |
| 15 | 58 | 81.0 | 35.1 | 0 | Metastasis | None | None | DAD | Grade 2 | 334 | 51 | Steroid Other | Yes |
| 16 | 64 | 45.0 | 18.5 | 1 | Metastasis | None | Pleural effusion | DAD | Grade 3 | 359 | 8 | Steroid | Yes |
| 17 | 77 | 52.0 | 22.5 | 1 | Recurrent | None | None | HP | Grade 1 | 67 | 7 | Steroid Other | Yes |

^a^All patients received T-DXd at a dose of 5.4 mg/kg except patient 16 who received 4.9 mg/kg of T-DXd

^b^T-DXd was interrupted in patient 13 and discontinued in the remaining patients after the onset of ILD

^c^No patient received a T-DXd rechallenge

^d^All patients were female

BMI, body mass index; CTCAE, Common Terminology Criteria for Adverse Events; DAD, diffuse alveolar damage; ECOG PS, Eastern Cooperative Oncology Group performance status; HP, hypersensitivity pneumonitis; ILD, interstitial lung disease; IS, immunosuppressant; NSIP, nonspecific interstitial pneumonia; OP, organizing pneumonia; T-DXd, trastuzumab deruxtecan

**Online Resource Supplementary Table 3** Univariate Cox regression analysis of factors for the development of ILD in patients treated with T-DXd for HER2-positive breast cancer (safety analysis set)

|  | **N** | **n (%)** | **95% CI** | **HR (95% CI)** |
| --- | --- | --- | --- | --- |
| **Sex** |  |  |  |  |
| Male | 8 | 4 (50.0) | 15.70–84.30 | 4.750 (1.769–12.754) |
| Female | 1,723 | 274 (15.9) | 14.21–17.72 | Reference |
| Unknown/missing | 0 | - |  | - |
| **Age, years** |  |  |  |  |
| <65 | 1,094 | 164 (15.0) | 12.93–17.25 | Reference |
| ≥65 | 637 | 114 (17.9) | 15.00–21.10 | 1.272 (1.002–1.616) |
| Unknown/missing | 0 | - |  | - |
| **Age, years** |  |  |  |  |
| <75 | 1,579 | 259 (16.4) | 14.61–18.32 | Reference |
| ≥75 | 152 | 19 (12.5) | 7.70–18.83 | 0.829 (0.520–1.322) |
| Unknown/missing | 0 | - |  | - |
| **Weight (kg)** |  |  |  |  |
| <40 | 130 | 17 (13.1) | 7.81–20.11 | 0.823 (0.504–1.344) |
| ≥40 | 1,594 | 261 (16.4) | 14.59–18.28 | Reference |
| Unknown/missing/not implemented | 7 | 0 (0.0) | 0.00–40.96 | - |
| **BMI (kg/m^2^)** |  |  |  |  |
| <21.3 (<Median) | 858 | 118 (13.8) | 11.52–16.24 | Reference |
| ≥21.3 (≥Median) | 858 | 158 (18.4) | 15.88–21.17 | 1.353 (1.066–1.717) |
| Unknown/missing | 15 | 2 (13.3) | 1.66–40.46 | 1.341 (0.331–5.429) |
| **BMI (kg/m^2^)** |  |  |  |  |
| <18.5 | 345 | 41 (11.9) | 8.66–15.78 | 0.702 (0.500–0.985) |
| ≥18.5–<25 | 1,096 | 183 (16.7) | 14.54–19.04 | Reference |
| ≥25 | 275 | 52 (18.9) | 14.46–24.05 | 1.090 (0.801–1.483) |
| Unknown/missing | 15 | 2 (13.3) | 1.66–40.46 | 1.089 (0.270–4.390) |
| **History of smoking** |  |  |  |  |
| Never smoked | 1,257 | 207 (16.5) | 14.46–18.64 | Reference |
| Current smoker+past smoker | 229 | 41 (17.9) | 13.17–23.49 | 1.155 (0.826–1.615) |
| Unknown/missing | 245 | 30 (12.2) | 8.42–17.02 | 0.777 (0.530–1.140) |
| **ECOG PS** |  |  |  |  |
| 0–1 | 1,594 | 260 (16.3) | 14.53–18.22 | Reference |
| 2–4 | 137 | 18 (13.1) | 7.98–19.97 | 1.209 (0.749–1.950) |
| Unknown/missing | 0 | - |  | - |
| **Time from recurrent or unresectable breast cancer diagnosis (months)** |  |  |  |  |
| <12 | 170 | 16 (9.4) | 5.48–14.83 | Reference |
| ≥12 | 1,541 | 259 (16.8) | 14.97–18.77 | 1.568 (0.946–2.598) |
| Unknown/missing | 20 | 3 (15.0) | 3.21–37.89 | 1.337 (0.390–4.590) |
| **Time from recurrent or unresectable breast cancer diagnosis (months)** |  |  |  |  |
| <60 | 1,203 | 183 (15.2) | 13.23–17.37 | Reference |
| ≥60 | 508 | 92 (18.1) | 14.86–21.74 | 1.153 (0.897–1.481) |
| Unknown/missing | 20 | 3 (15.0) | 3.21–37.89 | 0.922 (0.295–2.885) |
| **Unresectable or recurrent breast cancer stage** |  |  |  |  |
| IIIB+IIIC | 102 | 9 (8.8) | 4.11–16.09 | Reference |
| IV | 1,467 | 237 (16.2) | 14.31–18.14 | 1.937 (0.996–3.770) |
| Other | 161 | 31 (19.3) | 13.47–26.20 | 2.195 (1.045–4.609) |
| Unknown/missing | 1 | 1 (100.0) | 2.50–100.00 | - |
| **Hormone receptor expression status** |  |  |  |  |
| Positive | 1,029 | 164 (15.9) | 13.75–18.32 | Reference |
| Negative | 690 | 113 (16.4) | 13.69–19.35 | 1.010 (0.794–1.283) |
| Unknown/missing | 12 | 1 (8.3) | 0.21–38.48 | 0.467 (0.065–3.333) |
| **HER2 receptor expression status (IHC method)** |  |  |  |  |
| 3+ | 1,280 | 205 (16.0) | 14.05–18.14 | Reference |
| 2+ and 1+ and unknown and not done | 451 | 73 (16.2) | 12.91–19.92 | 1.094 (0.838–1.429) |
| **Lung/pleural metastasis or recurrence** |  |  |  |  |
| No | 837 | 113 (13.5) | 11.26–16.00 | Reference |
| Yes | 894 | 165 (18.5) | 15.96–21.16 | 1.375 (1.082–1.747) |
| **Liver metastasis or recurrence** |  |  |  |  |
| No | 1,122 | 199 (17.7) | 15.54–20.10 | Reference |
| Yes | 609 | 79 (13.0) | 10.41–15.90 | 0.749 (0.577–0.972) |
| **Brain metastasis or recurrence** |  |  |  |  |
| No | 1,364 | 210 (15.4) | 13.52–17.42 | Reference |
| Yes | 367 | 68 (18.5) | 14.69–22.89 | 1.210 (0.920–1.590) |
| **Prior cancer therapy for unresectable or recurrent breast cancer** |  |  |  |  |
| **Number of regimens** |  |  |  |  |
| <3 | 503 | 83 (16.5) | 13.36–20.04 | Reference |
| ≥3 | 1,212 | 192 (15.8) | 13.83–18.02 | 0.963 (0.745–1.246) |
| Unknown/missing | 16 | 3 (18.8) | 4.05–45.65 | 1.219 (0.385–3.858) |
| **Number of regimens** |  |  |  |  |
| <6 | 1,289 | 205 (15.9) | 13.95–18.02 | Reference |
| ≥6 | 426 | 70 (16.4) | 13.04–20.30 | 1.038 (0.791–1.362) |
| Unknown/missing | 16 | 3 (18.8) | 4.05–45.65 | 1.263 (0.404–3.950) |
| **Number of regimens** |  |  |  |  |
| <10 | 1,620 | 259 (16.0) | 14.23–17.86 | Reference |
| ≥10 | 95 | 16 (16.8) | 9.94–25.90 | 1.232 (0.744–2.042) |
| Unknown/missing | 16 | 3 (18.8) | 4.05–45.65 | 1.266 (0.406–3.950) |
| **Anti-HER2 therapies (Lapatinib)** |  |  |  |  |
| No | 1,174 | 184 (15.7) | 13.64–17.88 | Reference |
| Yes | 554 | 94 (17.0) | 13.93–20.36 | 1.037 (0.809–1.329) |
| Unknown/missing | 3 | 0 (0.0) | 0.00–70.76 | - |
| **Molecularly targeted therapies (any)** |  |  |  |  |
| No | 1,496 | 242 (16.2) | 14.34–18.14 | Reference |
| Yes | 232 | 36 (15.5) | 11.11–20.83 | 1.190 (0.838–1.689) |
| Unknown/missing | 3 | 0 (0.0) | 0.00–70.76 | - |
| **Molecularly targeted therapies (Bevacizumab)** |  |  |  |  |
| No | 1,560 | 252 (16.2) | 14.36–18.08 | Reference |
| Yes | 168 | 26 (15.5) | 10.37–21.85 | 1.183 (0.789–1.772) |
| Unknown/missing | 3 | 0 (0.0) | 0.00–70.76 | - |
| **Molecularly targeted therapies (Everolimus)** |  |  |  |  |
| No | 1,702 | 274 (16.1) | 14.38–17.93 | Reference |
| Yes | 26 | 4 (15.4) | 4.36–34.87 | 1.063 (0.396–2.851) |
| Unknown/missing | 3 | 0 (0.0) | 0.00–70.76 | - |
| **Molecularly targeted therapies (Palbociclib  or Abemaciclib)** |  |  |  |  |
| No | 1,654 | 269 (16.3) | 14.52–18.13 | Reference |
| Yes | 74 | 9 (12.2) | 5.71–21.84 | 1.005 (0.517–1.953) |
| Unknown/missing | 3 | 0 (0.0) | 0.00–70.76 | - |
| **Number of regimens for all prior treatments against breast cancer** |  |  |  |  |
| <10 | 1,476 | 234 (15.9) | 14.03–17.82 | Reference |
| ≥10 | 223 | 38 (17.0) | 12.35–22.63 | 1.151 (0.817–1.622) |
| Unknown/missing | 32 | 6 (18.8) | 7.21–36.44 | 1.451 (0.645–3.264) |
| **Prior chest radiation therapy (any)** |  |  |  |  |
| No | 925 | 138 (14.9) | 12.68–17.38 | Reference |
| Yes | 784 | 136 (17.3) | 14.76–20.18 | 1.218 (0.961–1.543) |
| Unknown/missing | 22 | 4 (18.2) | 5.19–40.28 | 1.281 (0.474–3.461) |
| **Prior chest radiation therapy (any adjuvant therapy)** |  |  |  |  |
| No | 1,174 | 186 (15.8) | 13.80–18.06 | Reference |
| Yes | 535 | 88 (16.4) | 13.41–19.87 | 1.083 (0.840–1.396) |
| Unknown/missing | 22 | 4 (18.2) | 5.19–40.28 | 1.197 (0.444–3.222) |
| **Prior chest radiation therapy (any recurrence therapy)** |  |  |  |  |
| No | 1,433 | 225 (15.7) | 13.85–17.69 | Reference |
| Yes | 276 | 49 (17.8) | 13.43–22.78 | 1.122 (0.823–1.528) |
| Unknown/missing | 22 | 4 (18.2) | 5.19–40.28 | 1.190 (0.443–3.199) |
| **Other prior and/or current medical history** |  |  |  |  |
| **ILD or radiation pneumonitis or COPD or emphysema or asthma** |  |  |  |  |
| No | 1,581 | 251 (15.9) | 14.11–17.77 | Reference |
| Yes | 150 | 27 (18.0) | 12.21–25.10 | 1.095 (0.736–1.628) |
| **ILD** |  |  |  |  |
| No | 1,697 | 266 (15.7) | 13.98–17.49 | Reference |
| Yes | 34 | 12 (35.3) | 19.75–53.51 | 2.272 (1.274–4.052) |
| **Radiation pneumonitis** |  |  |  |  |
| No | 1,661 | 267 (16.1) | 14.34–17.93 | Reference |
| Yes | 70 | 11 (15.7) | 8.11–26.38 | 0.890 (0.487–1.627) |
| **COPD or emphysema** |  |  |  |  |
| No | 1,726 | 277 (16.0) | 14.35–17.87 | Reference |
| Yes | 5 | 1 (20.0) | 0.51–71.64 | 2.257 (0.317–16.041) |
| **Asthma** |  |  |  |  |
| No | 1,680 | 273 (16.3) | 14.52–18.10 | Reference |
| Yes | 51 | 5 (9.8) | 3.26–21.41 | 0.610 (0.252–1.478) |
| **Pleural effusion** |  |  |  |  |
| No | 1,501 | 237 (15.8) | 13.98–17.73 | Reference |
| Yes | 230 | 41 (17.8) | 13.11–23.40 | 1.444 (1.036–2.012) |
| **History of lung surgery** |  |  |  |  |
| No | 1,655 | 259 (15.6) | 13.93–17.49 | Reference |
| Yes | 76 | 19 (25.0) | 15.77–36.26 | 1.643 (1.031–2.618) |
| **Pre-treatment laboratory tests and SpO_2_** |  |  |  |  |
| **Serum albumin (g/dL)** |  |  |  |  |
| Normal: ≥3.5 | 1,095 | 186 (17.0) | 14.81–19.34 | Reference |
| Mild: ≥3–<3.5 | 270 | 38 (14.1) | 10.16–18.80 | 1.047 (0.739–1.485) |
| Moderate+severe: <3 | 110 | 13 (11.8) | 6.45–19.36 | 1.099 (0.626–1.929) |
| Unknown/missing/not implemented | 256 | 41 (16.0) | 11.74–21.09 | 0.969 (0.691–1.359) |
| **SpO_2_ (%)** |  |  |  |  |
| <95 | 51 | 5 (9.8) | 3.26–21.41 | 0.756 (0.312–1.835) |
| ≥95 | 1,297 | 218 (16.8) | 14.81–18.96 | Reference |
| Unknown/missing/not implemented | 383 | 55 (14.4) | 11.00–18.28 | 0.835 (0.621–1.122) |
| **Renal impairment (determined by CrCL [mL/min])** |  |  |  |  |
| Normal renal function: ≥90 | 605 | 77 (12.7) | 10.18–15.65 | Reference |
| Mild renal impairment: ≥60–<90 | 767 | 141 (18.4) | 15.70–21.31 | 1.534 (1.162–2.025) |
| Moderate renal impairment+severe renal impairment+ESRD: <60 | 332 | 58 (17.5) | 13.54–21.99 | 1.493 (1.062–2.100) |
| Unknown/missing | 27 | 2 (7.4) | 0.91–24.29 | 0.707 (0.174–2.878) |
| **Hepatic impairment (determined by total bilirubin [mg/dL] and AST [IU/L])** |  |  |  |  |
| Normal hepatic function: TB ≤1.5 and AST ≤30 | 673 | 119 (17.7) | 14.87–20.78 | Reference |
| Mild dysfunction: TB >1.5–≤2.25 or AST >30 | 1,005 | 151 (15.0) | 12.87–17.39 | 0.895 (0.704–1.138) |
| Moderate dysfunction+severe dysfunction: TB >2.25 | 21 | 6 (28.6) | 11.28–52.18 | 2.133 (0.939–4.845) |
| Unknown/missing | 32 | 2 (6.3) | 0.77–20.81 | 0.347 (0.086–1.405) |

AST, aspartate aminotransferase; BMI, body mass index; CI, confidence interval; COPD, chronic obstructive pulmonary disease; CrCL, creatinine clearance; ECOG PS, Eastern Cooperative Oncology Group performance status; ESRD, end-stage renal disease; HER2, human epidermal growth factor receptor type 2; HR, hazard ratio; IHC, immunohistochemistry; ILD, interstitial lung disease; SpO_2_, oxygen saturation; TB, total bilirubin; T-DXd, trastuzumab deruxtecan

**Online Resource Supplementary Table 4** Multivariable Cox regression analysis of factors for the development of ILD in patients treated with T-DXd for HER2-positive breast cancer (safety analysis set)

|  | **N** | **n (%)** | **95% CI** | **HR (95% Cl)** |
| --- | --- | --- | --- | --- |
| **Sex** |  |  |  |  |
| Male | 8 | 4 (50.0) | 15.70–84.30 | 3.634 (1.299–10.163) |
| Female | 1,722 | 273 (15.9) | 14.16–17.67 | Reference |
| **Age (years)** |  |  |  |  |
| <65 | 1,094 | 164 (15.0) | 12.93–17.25 | Reference |
| ≥65 | 636 | 113 (17.8) | 14.87–20.96 | 0.945 (0.716–1.246) |
| **BMI (kg/m^2^)** |  |  |  |  |
| <21.3 (<Median) | 858 | 118 (13.8) | 11.52–16.24 | Reference |
| ≥21.3 (≥Median) | 857 | 157 (18.3) | 15.78–21.08 | 1.649 (1.275–2.133) |
| Unknown/missing | 15 | 2 (13.3) | 1.66–40.46 | 1.987 (0.465–8.499) |
| **ECOG PS** |  |  |  |  |
| 0–1 | 1,593 | 259 (16.3) | 14.48–18.16 | Reference |
| 2–4 | 137 | 18 (13.1) | 7.98–19.97 | 1.292 (0.781–2.137) |
| Unknown/missing | 0 | - |  | - |
| **Unresectable or recurrent breast cancer stage** |  |  |  |  |
| IIIB+IIIC | 102 | 9 (8.8) | 4.11–16.09 | Reference |
| IV | 1,467 | 237 (16.2) | 14.31–18.14 | 1.993 (1.012–3.928) |
| Other | 161 | 31 (19.3) | 13.47–26.20 | 2.080 (0.980–4.417) |
| Unknown/missing | 0 | - | - |  |
| **Lung/pleural metastasis or recurrence** |  |  |  |  |
| No | 837 | 113 (13.5) | 11.26–16.00 | Reference |
| Yes | 893 | 164 (18.4) | 15.88–21.06 | 1.227 (0.954–1.579) |
| **Liver metastasis or recurrence** |  |  |  |  |
| No | 1,121 | 198 (17.7) | 15.47–20.02 | Reference |
| Yes | 609 | 79 (13.0) | 10.41–15.90 | 0.742 (0.568–0.968) |
| **ILD** |  |  |  |  |
| No | 1,697 | 266 (15.7) | 13.98–17.49 | Reference |
| Yes | 33 | 11 (33.3) | 17.96–51.83 | 2.237 (1.210–4.134) |
| **Pleural effusion** |  |  |  |  |
| No | 1,500 | 236 (15.7) | 13.93–17.68 | Reference |
| Yes | 230 | 41 (17.8) | 13.11–23.40 | 1.340 (0.936–1.919) |
| **History of lung surgery** |  |  |  |  |
| No | 1,655 | 259 (15.6) | 13.93–17.49 | Reference |
| Yes | 75 | 18 (24.0) | 14.89–35.25 | 1.366 (0.829–2.252) |
| **Serum albumin (g/dL)** |  |  |  |  |
| Normal: ≥3.5 | 1,094 | 185 (16.9) | 14.73–19.27 | Reference |
| Mild: ≥3–<3.5 | 270 | 38 (14.1) | 10.16–18.80 | 0.973 (0.680–1.393) |
| Moderate and severe: <3 | 110 | 13 (11.8) | 6.45–19.36 | 0.961 (0.529–1.747) |
| Unknown/missing/not implemented | 256 | 41 (16.0) | 11.74–21.09 | 1.033 (0.730–1.461) |
| **SpO_2_ (%)** |  |  |  |  |
| <95 | 51 | 5 (9.8) | 3.26–21.41 | 0.665 (0.270–1.637) |
| ≥95 | 1,296 | 217 (16.7) | 14.75–18.89 | Reference |
| Unknown/missing/not implemented | 383 | 55 (14.4) | 11.00–18.28 | 0.870 (0.644–1.175) |
| **Renal impairment (determined by CrCL [mL/min])** |  |  |  |  |
| Normal renal function: ≥90 | 605 | 77 (12.7) | 10.18–15.65 | Reference |
| Mild renal impairment: ≥60–<90 | 766 | 140 (18.3) | 15.60–21.20 | 1.719 (1.272, 2.322) |
| Moderate renal impairment+severe renal impairment+ESRD: <60 | 332 | 58 (17.5) | 13.54–21.99 | 1.850 (1.240–2.761) |
| Unknown/missing | 27 | 2 (7.4) | 0.91–24.29 | 0.648 (0.149–2.819) |

BMI, body mass index; CI, confidence interval; CrCL, creatinine clearance; ECOG PS, Eastern Cooperative Oncology Group performance status; ESRD, end-stage renal disease; HER2, human epidermal growth factor receptor type 2; HR, hazard ratio; ILD, interstitial lung disease; SpO_2_, oxygen saturation; T-DXd, trastuzumab deruxtecan

**Online Resource Supplementary Table 5** ORR and BOR in patients treated with T-DXd (effectiveness analysis set)

|  | **Effectiveness analysis set (n=1,711)** | | **Patients with brain metastasis (n=365)** | |
| --- | --- | --- | --- | --- |
|  | **n (%)** | **95% CI** | **n (%)** | **95% CI** |
| **ORR^a^** | 1,036 (60.5) | 58.2–62.9 | 133 (36.4)^b^ | 31.5–41.4 |
| **BOR^c^** |  |  |  |  |
| **CR** | 98 (5.7) | **-** | 31 (8.5) | **-** |
| **PR** | 938 (54.8) | **-** | 102 (27.9) | **-** |
| **SD** | 448 (26.2) | **-** | 135 (37.0) | **-** |
| **PD** | 155 (9.1) | **-** | 20 (5.5) | **-** |
| **NE** | 72 (4.2) | **-** | 74 (20.3) | **-** |

^a^CR+PR

^b^IC-ORR in patients with brain metastasis

^c^IC-BOR for patients with brain metastasis

BOR, best overall response; CI, confidence interval; CR, complete response; IC, intracranial; NE, not evaluable; ORR, overall response rate; PD, progressive disease; PR, partial response; SD, stable disease; T-DXd, trastuzumab deruxtecan
